# Case report: *LMNB1* duplication-mediated autosomal dominant adult leukodystrophy in a Chinese family and literature review of Chinese patients

**DOI:** 10.3389/fnins.2025.1531593

**Published:** 2025-02-19

**Authors:** Yumeng Jiang, Lu Han, Yaqi Li, Zhihong Zhao, Zikai Xin, Zilong Zhu

**Affiliations:** ^1^Clinical College of Neurology, Neurosurgery, and Neurorehabilitation, Tianjin Medical University, Tianjin, China; ^2^Department of Electroencephalogram, Tianjin Huanhu Hospital, Tianjin, China; ^3^Department of Neurology, Tianjin Huanhu Hospital, Tianjin, China

**Keywords:** autosomal dominant adult leukodystrophy, *LMNB1* gene, Chinese family, whole exome sequencing, the multiplex ligation-dependent probe amplification, case report

## Abstract

Adult-onset autosomal dominant leukodystrophy (ADLD) is a rare, slowly progressive, and fatal neurodegenerative disorder characterized by central nervous system white matter loss due to *LMNB1* gene abnormalities encoding laminB1. However, not all *LMNB1* mutations lead to ADLD. Currently, two genetic alterations have been identified in association with the pathogenesis of ADLD: *LMNB1* gene tandem duplication and *LMNB1* gene upstream deletions. We report a case of a 60-year-old man diagnosed with ADLD, with pyramidal tract dysfunction and autonomic abnormalities as the main clinical manifestations. MRI revealed bilateral symmetric high signal intensities in the white matter of the medulla oblongata, middle cerebellar peduncles, cerebral peduncle, periventricular white matter, centrum semi vale, and the pressure region of the corpus callosum. Whole exome sequencing results indicated 73.6Kb duplicate copy number variation signals in the 5q23.2 region of the proband’s chromosome. The Multiplex ligation-dependent probe amplification (MLPA) experiment results indicate recurrent mutations across all exons (exon1–11) of the *LMNB1* gene. This is the eighth ADLD pedigree from China. We conducted a literature review of all ADLD pedigrees in China and summarized the characteristics of Chinese patients with ADLD to raise awareness of ADLD disease.

## 1 Introduction

Adult-onset autosomal dominant leukodystrophy (ADLD) is a rare, slowly progressive, and fatal neurodegenerative disorder characterized by the loss of central nervous system (CNS) white matter (Neri et al., 2023), caused by either *LMNB1* duplications or heterozygous deletions upstream of the *LMNB1* promoter (Padiath et al., 2006; Giorgio et al., 2015). Both distinct mechanisms result in overexpression of *LMNB1* and abnormal intracellular accumulation, contributing to selective progressive CNS demyelination in a way, but the specific pathological mechanisms remain unclear (Rolyan et al., 2015; Padiath, 2019). All reported Chinese families of ADLD are due to *LMNB1* gene duplication. The *LMNB1* gene, situated at chromosome 5q23.2, encodes the protein laminB1.Overexpression of *LMNB1* protein in ADLD has been associated with increased nuclear rigidity in fibroblasts and dysregulation of alternative RNA splicing, affecting RNA splicing processes (Ferrera et al., 2014; Bartoletti-Stella et al., 2015). Studies also have found that overexpression of *LMNB1* may lead to oligodendrocyte dysfunction and subsequent demyelination (Rolyan et al., 2015; Lin and Fu, 2009). Furthermore, *LMNB1* overexpression can decrease the expression of lipid synthesis genes and myelin-enriched lipids via age-dependent epigenetic modifications (Rolyan et al., 2015; Padiath, 2016), partially accounting for the late onset. Recent studies indicate that *LMNB1* overexpression may impair astrocytic function, diminishing their essential support to oligodendrocytes during myelination (Ratti et al., 2021a,b). Non-myelinating cells are vulnerable to *LMNB1* overexpression, potentially playing a role in ADLD pathogenesis (Roy et al., 2023). The LMNB1 gene and its association with the disease is given in Supplementary Figure S1. ADLD is the sole central nervous system demyelinating disease linked to *LMNB1* overexpression (Neri et al., 2023; Padiath and Fu, 2010), first identified by Eldridge et al. (1984).

Up to now, over 30 families have been diagnosed globally, but precise prevalence data remain unavailable (Raininko et al., 1993). Unlike most hereditary leukodystrophies that usually present in infancy or early childhood, ADLD typically manifests with classical clinical symptoms in the fourth or fifth decade of life (Coffeen et al., 2000). Affected individuals generally survive for one to two decades following clinical onset (Finnsson et al., 2015). Initial clinical symptoms frequently include autonomic abnormalities like bladder dysfunction, constipation, orthostatic hypotension, erectile dysfunction, and impaired sweating. Pyramidal tract and cerebellar involvement typically appear months to years later, resulting in spastic weakness of extremities, gait ataxia, nystagmus, dysmetria, intention tremor, etc. (Padiath and Fu, 2010). In the early stages of the disease, cognitive function is typically maintained or only slightly impaired, but cognitive decline and psychiatric issues may arise in the later stages (Raininko et al., 1993). MRI of the brain and spinal cord can detect diffuse, confluent, and symmetrical white matter lesions. An ADLD Chinese family with autonomic abnormalities and pyramidal tract dysfunction is presented, and to date, a total of 7 Chinese families with ADLD have been reported. We conducted a literature review of these case reports and observed the clinical characteristics of these Chinese families.

## 2 Clinical data

The participant originated from a northern Chinese family. All participants provided written informed consent for the publication of this case report. The study adhered to the Declaration of Helsinki and relevant Chinese policies.

## 3 Case presentation

**Figure 1:**
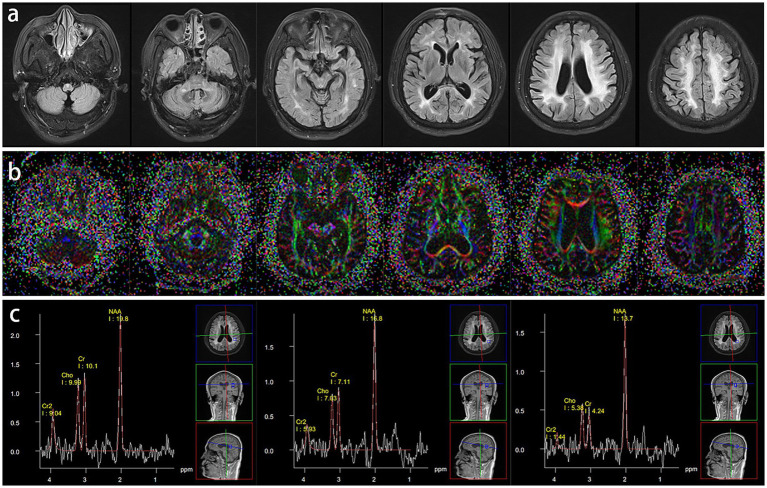
Brain MRI **(A)**, DTI **(B)**, and MRS **(C)** of the proband.

The proband (II_2_) developed gait disturbances at age 54, which began with occasional foot-dragging while walking, and later developed spastic weakness in both lower limbs after prolonged walking. Worsening at age 58, he walked slowly and laboriously and needed support against the wall after walking short distances. Simultaneously, He began to develop a feeling of weakness in both upper limbs, accompanied by episodic dizziness and a top-heavy sensation. Autonomic symptoms, including sleep disorders, frequent urination, and constipation, appeared prior to pyramidal tract dysfunction. Neurological examination of the proband showed both lower limbs muscle strength of grade 4 and both upper limbs muscle strength of grade 5, mild muscular tension, brisk tendon reflexes, positive pathological reflexes, no cerebellar ataxia, and cognitive impairment. His resting blood pressure was 150/94 mmHg, but postural hypotension was not assessed at that time. The proband’s brain MRI revealed symmetric confluent long T2 signals in the medulla oblongata, middle cerebellar peduncles, cerebral peduncle, periventricular regions, centrum semi-oval, and corpus callosum (Figure 1A). Diffusion tensor magnetic resonance imaging (DTI) showed that the affected area of fiber bundles corresponded with MRI findings, with decreased fractional anisotropy (FA) values (Figure 1B). Magnetic resonance spectroscopy (MRS) revealed minor reductions in N-acetyl aspartate (NAA), choline (Cho), and creatine (Cr) within the lesion area relative to normal regions. The Cho/NAA ratio did not increase and no obvious proliferative changes were observed (Figure 1C).

The proband’s father (I_1_) also had similar gait disturbances for over 10 years, lacking a clear diagnosis or special treatment, and died of lung cancer at about 70 years old at last. His son (III_1_) had similar foot-dragging symptoms while walking, but did not undergo MRI evaluation. Relevant physical examinations have not revealed any positive signs, which may be related to his age, and regular follow-up observations are required. His nephew (III_5_) was diagnosed with white matter demyelinating lesions in other hospitals, highly suspected to be ADLD, but no significant clinical manifestations were observed and genetic testing was not undergone. His other relatives (II_4_, II_5_, III_3_, IV_2_) are asymptomatic and have not been examined.

## 4 Genetic tests and treatment

**Figure 2:**
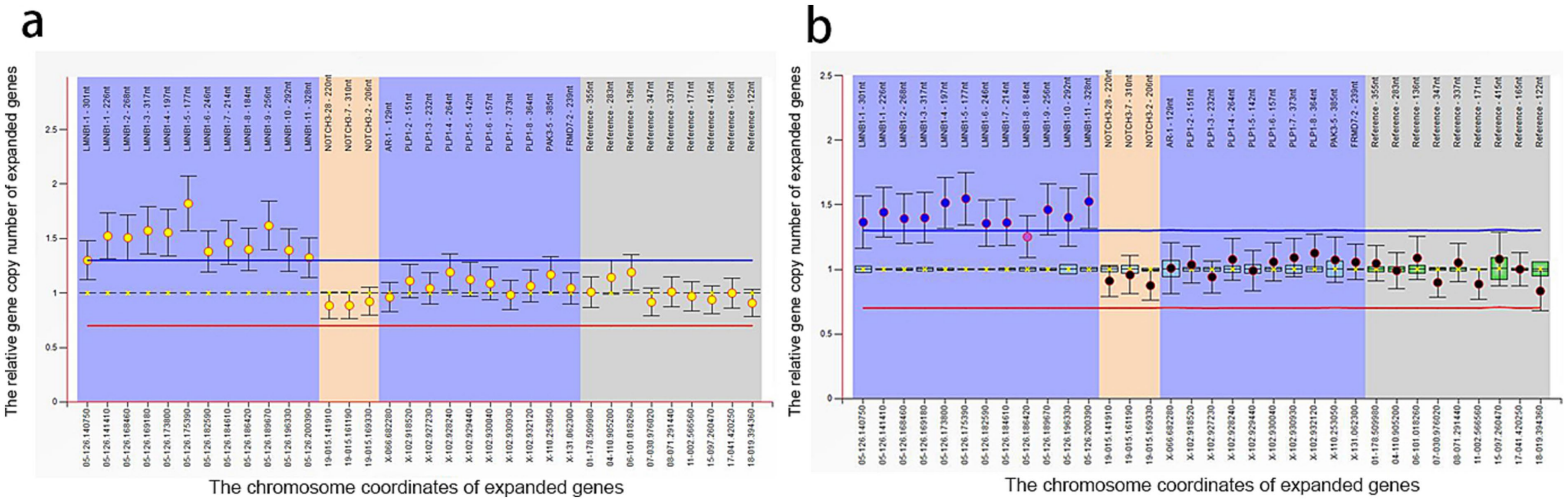
Multiplex ligand-dependent probe amplification experiment of II_1_ **(A)** and III_1_ **(B)**.

**Figure 3:**
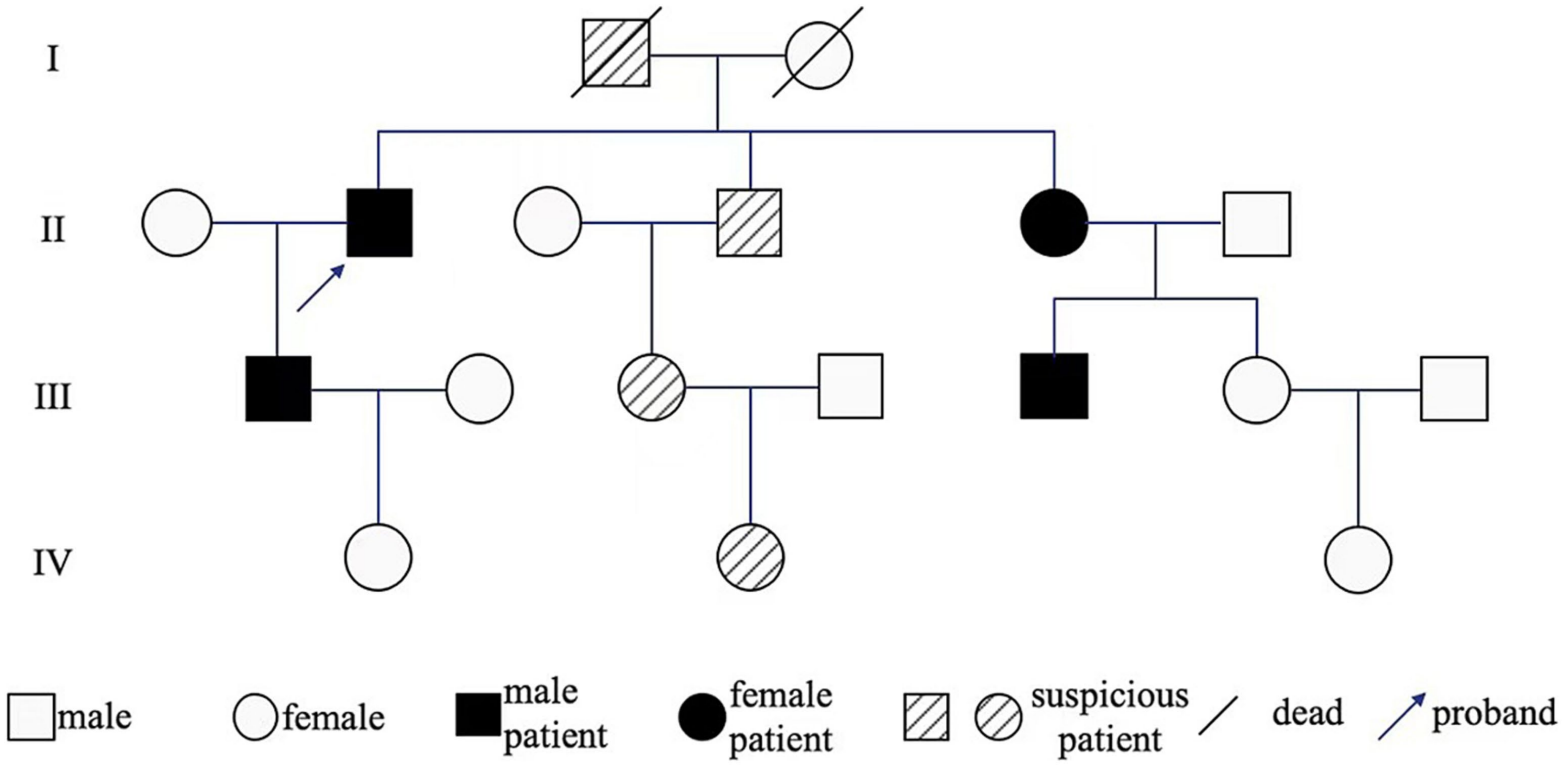
Genealogy map of ADLD. I_1_ developed gait disturbances in middle and old age and died of lung cancer at age 70; II_2_ constipation, gait disturbances appeared and gradually worsened at 54 years old; III_1_ occasional foot-dragging while walking; II_4_, II_5_, III_3_, IV_2_ are asymptomatic and have not been examined; III_5_ MRI examination revealed white matter demyelinating lesions, suggesting that II_5_ carried *LMNB1* mutated gene.

Peripheral blood samples from the proband (II_1_) were collected for whole exome sequencing (WES) of genomic DNA. Peripheral blood DNA of the family members was extracted using the QIamp DNA Blood Mini Kit (Qiagen, Hilden, Germany). Whole exome capture for the proband’s DNA sample was performed using the xGen Exome Research Panel from Integrated DNA Technologies (Integrated DNA Technologies, Skokie, USA). The sequencing was conducted on the Illumina Novaseq 6,000 platform, with an average target sequencing depth of 200x. After filtering, the clean reads were aligned to the human reference genome (GRCh37, hg19) using BWA-MEM. Variant calling was guided by the GATK Best Practices. Variants with the “PASS” mark and coverage reads ≥20 were annotated using ANNOVAR integrated with available databases, such as RefSeq Gene, dbSNP150, ClinVar, and allele frequencies in populations from 1000G, ESP6500, and ExAC database. After the causative variants of the probands were found, Sanger sequencing was applied for the family members. Results indicated 73.6Kb duplicate copy number variation signals in the 5q23.2 region of the proband’s chromosome. The duplicate copy number variation region primarily contained *LMNB1* and MARCHF3 genes on the genomic DNA of the patient. The Multiplex ligation-dependent probe amplification (MLPA) experiment results indicate recurrent mutations across all exons (exon1–11) of the *LMNB1* gene. No duplication or deletion was found in NOTCH3 and PLP1 genes (Figure 2A). Therefore, he was diagnosed with *LMNB1* duplication-mediated ADLD. The MLPA results of the proband’s son (III_1_) matched his father’s, also with recurrent mutations in the *LMNB1* gene (Figure 2B). Unfortunately, further genetic testing was not performed on the proband’s other relatives. The family pedigree is given in Figure 3. Effective treatment strategies for ADLD are missing. These patients were treated with symptomatic therapy only.

## 5 Discussion

In our study, we confirmed the eighth pedigree diagnosed with ADLD in China and summarized the clinical and imaging features of all ADLD pedigrees in the country (Table 1) (Dai et al., 2017; Zhang et al., 2019; He et al., 2021; Chen et al., 2022; Chen et al., 2023; Si-qi and Heng, 2023; Song et al., 2024). Five patients started with autonomic abnormalities and pyramidal signs, of which one patient reported pyramidal signs prior to autonomic abnormalities, which is consistent with typical ADLD onset mode but suggests potential individual differences. Varying degrees of cognitive impairment were observed in five patients, possibly related to disease progression. Two patients manifested double upper limb tremors, with one identifying tremor as the initial symptom. Combined with a head MRI, it could be inferred that the lesion involved the cerebellum. Two patients experienced sudden disturbance of consciousness and poor prognosis. It is reported that heat intolerance and false deterioration can appear in ADLD patients, manifesting as cognitive decline, disturbance of consciousness, and motor symptoms, but these changes are often reversible with disease improvement (Finnsson et al., 2015), and the disturbance of consciousness may also be associated with epilepsy. One patient presented with rare transient hypoglycemia and unilateral pupil dilation, potentially related to autonomic dysfunction caused by sympathetic nerve injury and adrenal medullary dysfunction (Terlizzi et al., 2016). All reported Chinese families are classic ADLD, but the cause of this feature remains unclear. This may be attributed to the rarity of ADLD, limiting its representativeness in the broader population, or it may reflect potential ethnic and genetic differences. Further global cases are needed to draw definitive conclusions. Non-classical clinical cases of *LMNB1* upstream deletions reported in other countries have demonstrated late onset, lacking autonomic dysfunction and cerebellar ataxia (Mezaki et al., 2018).

**Table 1:** Eight ADLD patients reported in China.

Family	Age	First symptoms	Major/other symptoms	Brain MRI lesions	Myelopathy	References
1	49	Autonomic abnormalities	Mild cognitive impairment	Centrum semi-oval, periventricular white matter, pyramidal tract, middle cerebellar peduncles		Dai et al. (2017)
2	58	Tremor	Autonomic abnormalities, pyramidal tract sign/cerebellar ataxia	Centrum semi-oval, corpus callosum, midbrain, pons, middle cerebellar peduncle	Diffuse atrophy	Zhang et al. (2019)
3	46	Autonomic abnormalities, pyramidal tract sign	Disturbance of consciousness/ transient hypoglycemia and unilateral pupil dilation, tremor, cerebellar ataxia	Centrum semi-oval, periventricular white matter, corpus callosum, cerebral peduncles, cerebellar peduncles	Cervical and thoracic cord atrophy	He et al. (2021)
4	54	Autonomic abnormalities, pyramidal tract sign	Cerebellar ataxia, mild cognitive impairment	Centrum semi-oval, periventricular white matter, corpus callosum, cerebellum, midbrain	Total spinal cord atrophy	Chen et al. (2022)
5	54	Pyramidal tract sign	Autonomic abnormalities, cognitive impairment	Centrum semi-oval, periventricular white matter, middle cerebellar peduncles		Chen et al. (2023)
6	25	Disturbance of consciousness	Pyramidal tract sign, cerebellar ataxia, autonomic abnormalities, cognitive impairment	Centrum semi-oval, periventricular white matter, thalamus, brainstem		Si-qi and Heng (2023)
7	41	Autonomic abnormalities, pyramidal tract sign	Cerebellar ataxia, cognitive impairment	Centrum semi-oval, middle cerebellar peduncles, brainstem	Thoracic atrophy	Song et al. (2024)
8	54	Autonomic abnormalities	Pyramidal tract sign	Centrum semi-oval, periventricular white matter, corpus callosum, cerebral peduncle, middle cerebellar peduncles, medulla oblongata		

MRI scanning is crucial for diagnosing ADLD, marked by diffuse, symmetrical leukodystrophy mainly affecting the frontal and parietal lobes, cerebellum, corpus callosum, and spinal cord, with minimal impact on periventricular white matter (Melberg et al., 2006; Sundblom et al., 2009). MRI abnormalities in the brain and spinal cord may emerge decades before clinical symptoms, with the extent of lesions correlating with the disease’s duration and severity (Finnsson et al., 2015; Zanigni et al., 2015). Notably, spinal cord MRI abnormalities can be obvious even when brain MRI changes are slight (Sundblom et al., 2009). In contrast to patients with classical ADLD, MRI findings in patients with nontypical ADLD showed selective white matter involvement, with less involvement in the cerebellum and medulla oblongata (Dimartino et al., 2024) (Supplementary Figure S2). Unfortunately, a spinal cord MRI was not performed on the proband. The imaging features of cases from China are consistent with those cases in other countries.

The onset age of ADLD is late, the disease progression is relatively slow. Early MRI examination and genetic detection can facilitate early intervention in the treatment of the disease. However, there is currently no effective treatment for ADLD, and any existing therapies primarily address clinical symptoms (Raininko et al., 1993). For example, the eight Chinese patients mentioned received dietary guidance, neurotrophic support, spasticity relief, functional exercise, cognitive improvement, and other treatments according to clinical symptoms. Recent studies suggest that reducing *LMNB1* levels and restoring small molecules associated with nuclear abnormalities may prevent the occurrence and progression of the disease (Lin and Fu, 2009; Lin et al., 2014; Nmezi et al., 2020), and protein regulation may provide new therapeutic opportunities for ADLD (Giorgio et al., 2021).

## 6 Conclusion

ADLD is a rare white matter disease of the central nervous system that progresses slowly and is inherited in an autosomal dominant manner. The possibility of *LMNB1* gene-related ADLD should be considered when a patient presents with slow progressive limb weakness, especially spastic weakness and autonomic dysfunction, combined with MRI suggesting symmetric leukodystrophy and family members have similar clinical symptoms. A comprehensive family history and thorough physical examination are crucial. Timely diagnosis and intervention can slow disease progression. This study has enhanced our understanding of ADLD by summarizing the clinical and imaging features of the eight currently identified families with ADLD in China, which is helpful for the early identification of the disease through clinical symptoms and MRI findings.

## Data Availability

The original contributions presented in the study are included in the article/Supplementary material, further inquiries can be directed to the corresponding author/s.
