# Supplementary material for: Case report: LMNB1 duplication-mediated autosomal dominant adult leukodystrophy in a Chinese family and literature review of Chinese patients
Source: Front Neurosci. 2025 Feb 19;19:1531593. doi: 10.3389/fnins.2025.1531593 (PMC11880262; doi:10.3389/fnins.2025.1531593)
Supplement: Supplementary file 1 [file Data_Sheet_1.DOCX]

Supplementary Figure


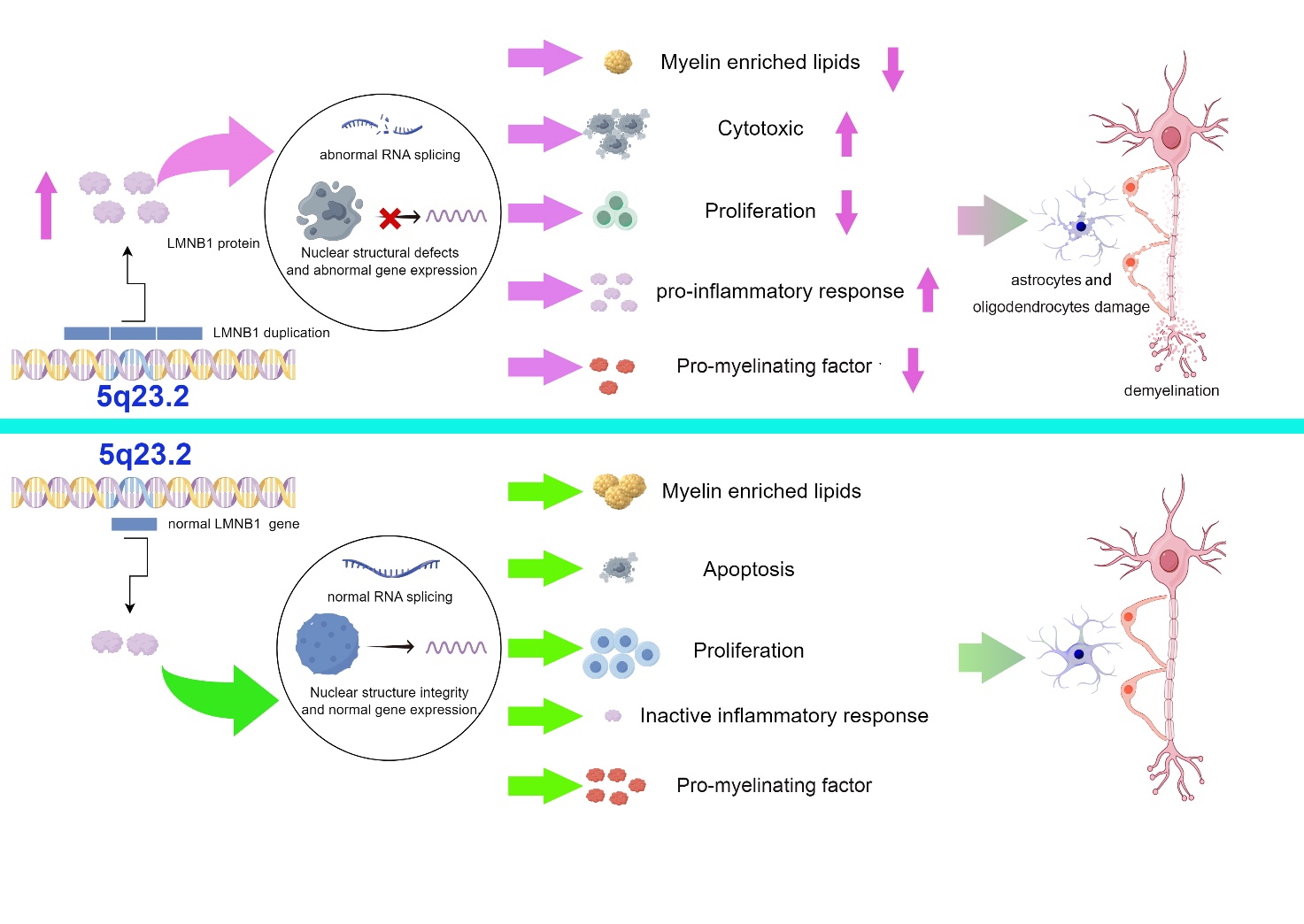


**Supplementary Figure 1.** The *LMNB1* gene and its possible pathogenic mechanisms.


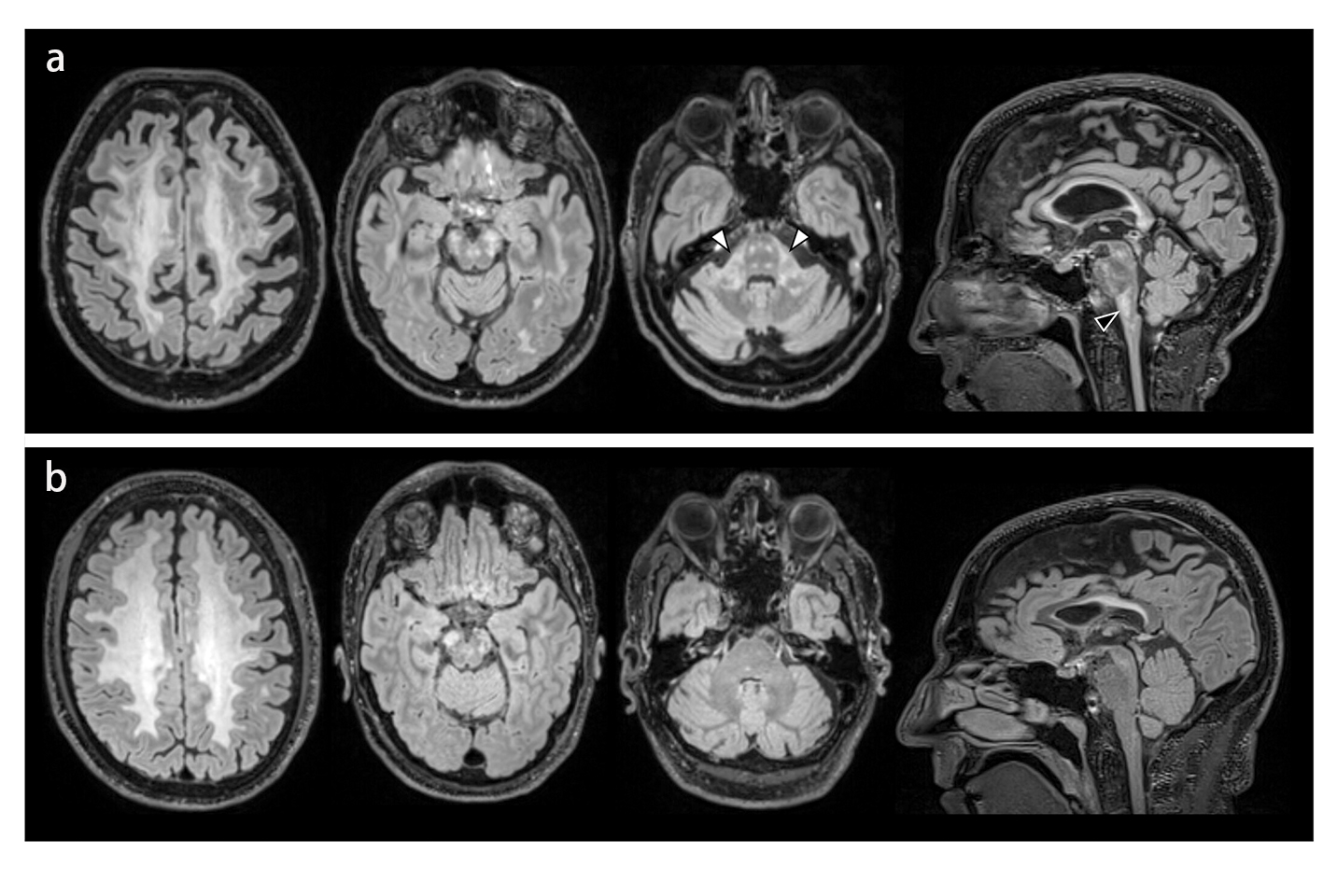
 **Supplementary Figure 2.** Representative brain MRI manifestations in patients with classic (a) and nontypical (b) ADLD. Dimartino, P., et al., Structural Variants at the LMNB1 Locus: Deciphering Pathomechanisms in Autosomal Dominant Adult-Onset Demyelinating Leukodystrophy. Ann Neurol, 2024.
